# An urgent need for community lot testing of lateral flow fentanyl test strips marketed for harm reduction in Northern America

**DOI:** 10.1186/s12954-024-01025-7

**Published:** 2024-06-15

**Authors:** Marya Lieberman, Adina Badea, Charlie Desnoyers, Kathleen Hayes, Ju Nyeong Park

**Affiliations:** 1 University of Notre Dame, Notre Dame, IN USA; 2 Lifespan Academic Medical Center, Providence, RI USA; 3 Brown University, Providence, RI USA; 4 Warren Alpert Medical School, Brown University, Providence, RI USA

**Keywords:** Drug supply, Fentanyl test strips, Validation

## Abstract

**Background:**

Fentanyl test strips (FTS) are lateral flow immunoassay strips designed for detection of ng/mL levels of fentanyl in urine. In 2021, the US Centers for Disease Control and the Substance Abuse and Mental Health Administration stated that federal funds could be used for procurement of FTS for harm reduction strategies approved by the government such as drug checking. The market for FTS has expanded rapidly in the US and Canada. However, there is no regulatory oversight by either government to ensure proper function of FTS that are being marketed for drug checking.

**Main body:**

Many brands of FTS have rapidly entered the harm reduction market, creating concerns about the reproducibility and accuracy of their performance from brand to brand and lot to lot. Some examples are provided in this Comment. Similar problems with product quality were observed in the mid 2000’s when lateral flow immunoassays for malaria were funded in many countries and again in 2020, when COVID-19 tests were in huge demand. The combination of high demand and low levels of regulation and enforcement led some manufacturers to join the goldrush without adequate field testing or quality assurance. We argue that the harm reduction community urgently needs to set a lot checking program in place. A set of simple protocols for conducting the tests and communicating the results have been developed, and are described in the following Perspectives paper in this issue.

**Conclusion:**

In the absence of governmental regulation and enforcement, the harm reduction community should implement a FTS lot checking program. Based on previous experience with the malaria diagnostic lot checking program, this inexpensive effort could identify products that are not suitable for harm reduction applications and provide valuable feedback to manufacturers. Dissemination of the results will help harm reduction organizations to ensure that FTS they use for drug checking are fit for the purpose.

## Background

US drug overdose deaths claimed 107,622 lives in 2021 and 105,452 in 2022 [1]. Most deaths involved illicitly-manufactured fentanyl, which has spread to every state in the US [2]. One strategy to reduce the harm caused by drug overdoses is drug checking. Drug checking involves point-of-use identification of components of illicitly obtained drugs, particularly components like fentanyl that are associated with increased risk of overdose. As part of activities directed at reducing the toll of opioid overdoses, many harm reduction organizations (HROs) have started fentanyl test strip (FTS) programs. FTS are immunoassay test strips that can rapidly detect fentanyl at 200 ng/mL concentration in water. FTS programs are useful for communities at risk, and demand for FTS is growing rapidly in the US and Canada [3–7]. In April of 2021, the US Substance Abuse and Mental Health Services Administration (SAMHSA) and the Centers for Disease Control and Prevention (CDC) issued approval for Federal grantees to use grant funds to purchase FTS for drug checking applications [8]. Over 8 million FTS from a single distributor were distributed by HROs in 2022, and in 2024, 16 branded products were readily identifiable from a quick search of one major online shopping site [9].

This rapid uptake of FTS by drug checking programs is occurring with no regulatory oversight of the quality of the FTS products. Lateral flow strips intended for use in monitoring fentanyl in urine are regulated by the US Food and Drug Administration (FDA), or by the Health Products and Food Branch of Canada, but neither agency has assessed the utility of any urinary FTS for use in drug checking or issued standards for this application [10]. Weak or absent regulation coupled with high demand is a known scenario that can lead to poor quality products gaining foothold in a market [11]. The harm reduction community is particularly concerned about inconsistencies between different strips and false positive or false negative drug checking results. For commonly used brands of FTS, studies have shown false positive rates for testing of street drugs in the range of 5-10.9% and false negative rates in the range of 1.5–12.5% [12, 13]. Several reasons for this range of performance have been documented, including inaccurate field sample preparation, sample heterogeneity, and off-target binding of the antibody to fentanyl analogs and other substances [14]. The influence of lot-to-lot variability of the FTS was not examined in these studies.

In this Commentary, we present evidence that brand-to-brand and lot-to-lot variability of FTS can cause inconsistent results on samples relevant for drug checking applications, and we propose a community lot quality checking program to proactively monitor FTS products that are marketed for drug checking purposes. The following Technical Note describes a proposed protocol for carrying out lot checking and reporting the results to a central dissemination site.

## Lot-to-lot variability and its causes

**Fig. 1:**
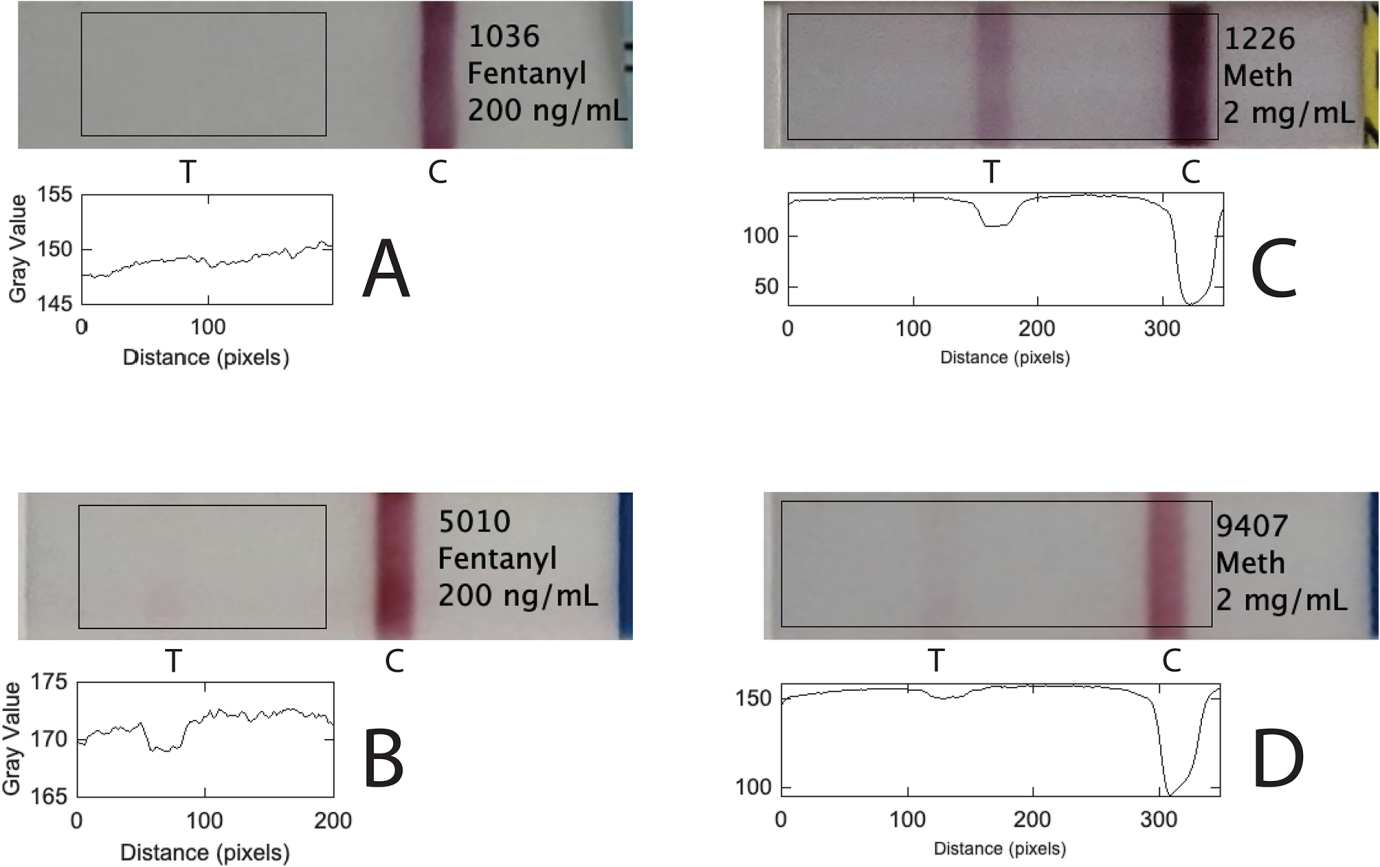
Brand to brand variability of test strip results. The plot profiles in the areas outlined by black rectangles were measured with ImageJ [15]. (**A**) WHPM lot F122221036 Fen10 shows no visible test line and therefore a positive result with 200 ng/mL fentanyl, while (**B**) BTNX lot D805010 shows a faint pink test line and therefore a negative result with 200 ng/mL fentanyl. 200 ng/mL is well above the stated LOD of fentanyl for each product (the LODs are stated for a urine matrix, not a water matrix). (**C**) WHPM lot K2051226 shows a strong pink color on the test line and therefore a negative result with 2 mg/mL methamphetamine HCl, while (**D**) BTNX lot DOA2109407 still shows a negative result with 2 mg/mL methamphetamine HCl, but the test line color is very weak and might be mistaken for a positive result

Fentanyl test strips are competitive immunoassays, which means that fentanyl prevents binding of colored conjugate material to the test line but not to the control line. While the control line should always turn pink or red, manufacturer instructions say that any color in the test line, even if it is very faint, should be counted as a negative result. Figure 1 illustrates how different brands of FTS can respond in different ways to the same sample. Figure 1A and B show results from two brands of FTS for a true positive sample, 200 ng/mL fentanyl in water. The plot profile beneath each strip shows the integrated pixel intensity along the strip. Figure 1A shows a clear positive result, but Fig. 1B shows a visible pink color on the test line. Some people might read Fig. 1B as a positive result, and others as a negative result. Figure 1C and D show results for two brands of FTS for methamphetamine at 2 mg/mL; Fig. 1C shows a strong negative result, but for Fig. 1D, the test line is very faint. Some people might read Fig. 1D as a positive result, and others as a negative result.

**Fig. 2:**
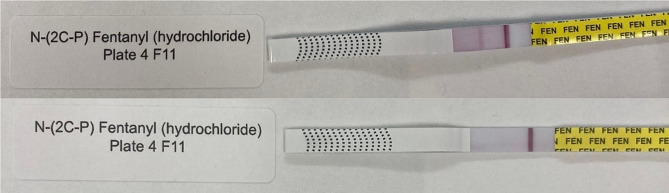
Lot to lot variability: Top: DanceSafe WHPM lot K2051226 gives negative result; Bottom: WHPM lot K2021217 gives positive result. N-(2 C-P)-Fentanyl at 2,000 ng/mL

**Fig. 3:**
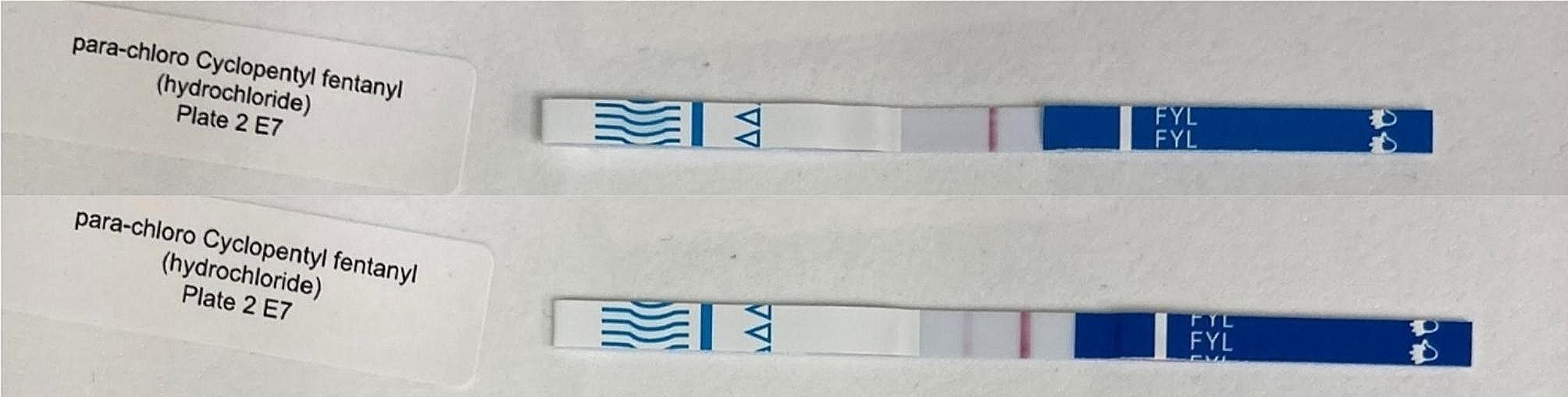
Lot to lot variability: Top: BTNX lot 2,204,104 gives positive result; Bottom BTNX lot 2,206,394 gives negative result. *Para*-chloro Cyclopentyl fentanyl at 20,000 ng/mL

Figures 2 and 3 show examples of lot-to-lot variability for detection of fentanyl analogs. This variability may be due to use of different fentanyl hapten/antibody pairs on the different strips [16].

**Fig. 4:**
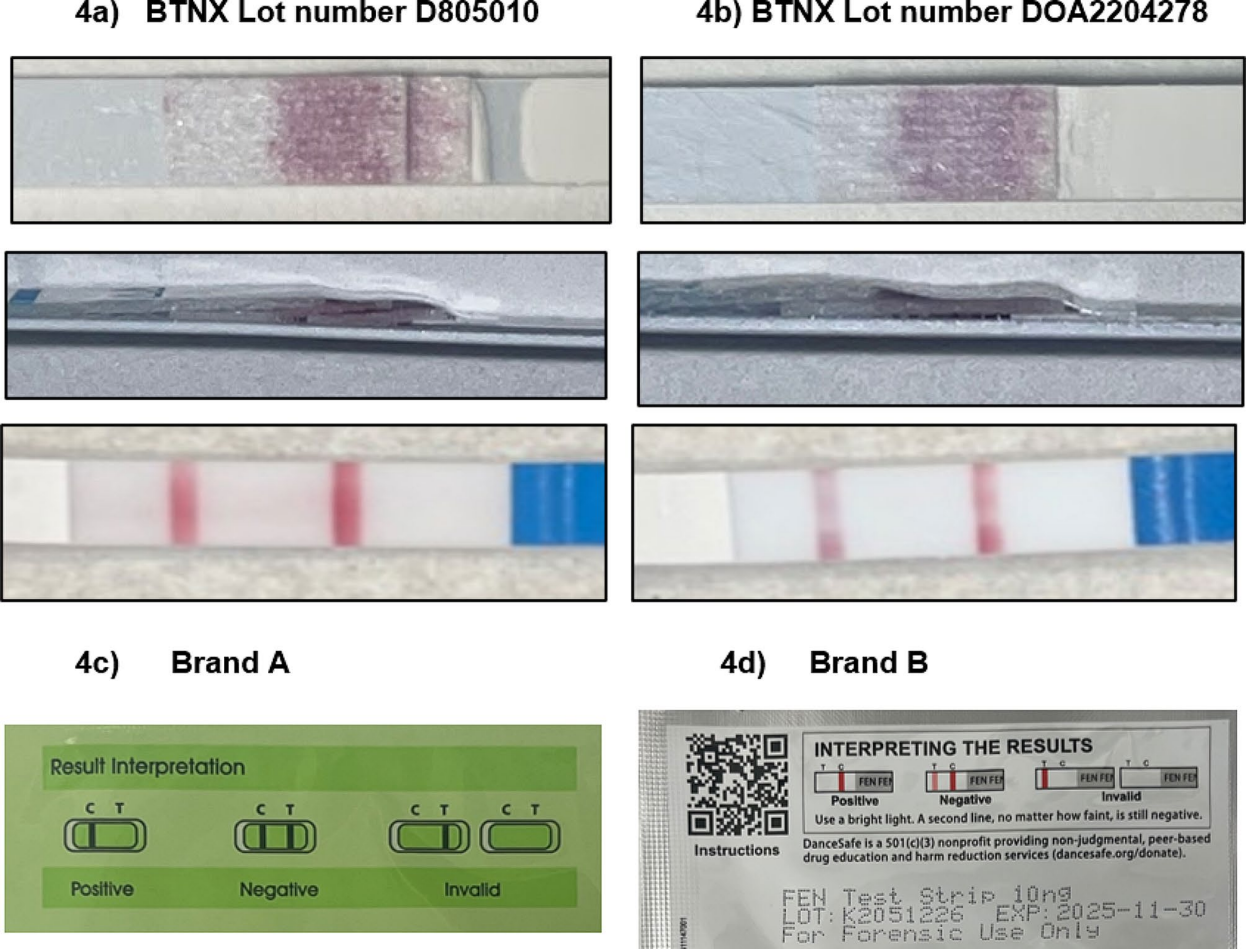
Variation of test strip construction and packaging. (**a**) Two conjugate pads; (**b**) one conjugate pad. In both **a**) and **b**), the top image shows the strip after removal of the plastic cover, the middle image shows a side view of the strip, and the bottom view shows the color intensity of the control and test lines after the strip is run in water (negative control). (**c**) and (**d**) result interpretation guidelines appear to show different locations of control and test lines, but in both strips, the control line is actually located further from the sample application pad than the test line. For **c**), the strip must be held upside-down to read it. For **d**), the strip is held right side up

FTS are a complex manufactured product. The manufacturing process involves assembly of sample application pad, conjugate pad, nitrocellulose membrane, and wicking pad on a plastic backing card, selection of monoclonal antibodies that can bind the anticipated target molecule and haptens that mimic the target molecule, controlled deposition of antibodies and/or haptens to delineate control and test lines, and blocking of the nitrocellulose to prevent non-specific binding [17]. Lot-to-lot variability can be caused by variations in any of the input materials, variations in the way the solutions are applied and dried, and differences in the visualizing agents or the monoclonal antibodies. Variability can also arise from differences in packaging and storage conditions of the finished strips or from differences in product design and labeling. Figure 4 shows construction details for two lots of a single brand of drug test strip. One lot uses two conjugate pads (Fig. 4a), the other lot has just one conjugate pad (Fig. 4b). The use of one vs. two conjugate pads is associated with lower color intensity in the test and control lines of the strips. Figure 4 also shows the packages from two brands of FTS. In both types of FTS, the control line is located farther from the sample loading pad than the test line. However, in one strip (Fig. 4c) the directions show the test line to the right of the control line, which means the strip must be held with the writing on the strip upside-down to compare the lines with the instructions. If the strip is not held with this orientation the user could interpret a positive test result as a strip failure. The instructions for the other strip (Fig. 4d) show a more natural orientation of the test strip and includes printed text (“FEN FEN”) which corresponds to text on the plastic cover of the strip as a further guide so the user holds the strip correctly. More recent packaging materials for Brand A have corrected this issue, indicating that manufacturers are receptive to suggestions from their customer base.

## Those who will not learn from history are doomed to repeat it…the case of malaria RDTs

The quality issues now arising with FTS have a historical precedent. In the mid 2000s, as part of the global effort to reduce malaria deaths in sub-Saharan Africa, hundreds of millions of rapid diagnostic tests (RDTs) per year were purchased by anti-malaria programs. Over 180 manufacturers joined the gold rush. Critical quality issues with some brands of RDTs rapidly became apparent [18]. Many brands of RDTs had unacceptable rates of false positive or false negative results, confusing or illegible instructions, or poor lot-to-lot reproducibility [19]. Similar issues were observed in 2020 for rapid diagnostics for COVID-19, [20] and these problems continue to date [21].

In 2007, a global lot testing program for RDTs, supported by the Foundation for Innovative New Diagnostics (FIND) and WHO was implemented [22, 23]. The testing protocol included defined positive and negative samples, workflows for lot testing, and proficiency testing for the technicians. At the program’s peak, 700 lots were tested each year. The estimated cost of testing each lot was just $255 USD [24]. Detailed feedback was reported to manufacturers so they could improve their products. The lot testing program was highly effective in improving the quality of malaria RDTs. **After six years of regular lot testing, the failure rate for the RDTs dropped from 29 to 0.18%**. The success of this program has informed WHO recommendations for post-market surveillance of other lateral flow devices, such as HIV diagnostic kits [25].

## Conclusions: the harm reduction community needs a lot checking program for FTS

There is currently strong economic incentive for new brands and lots of FTS to enter the drug-checking market, and evidence that brand-to-brand and lot-to-lot variability can cause confusing or incorrect results. In order to make sure that the FTS used in harm reduction programs are accurate, reliable, and fit for the purpose of drug checking, we are starting a community program to proactively monitor the quality of FTS that are being marketed for harm reduction. Details of the standard operating protocol (SOP) for lot checking are provided in the accompanying article [26]. We anticipate the cost per lot should be well under $250, including materials and researcher time. Our goal is to identify problematic lots of FTS for the community using the FTS and for the manufacturers and distributors of these products. Preliminary data can be viewed at https://tinyurl.com/LotResults. We hope this project will set a foundation for monitoring other drug testing products, such as xylazine or benzodiazepine test strips. Readers who are interested in participating in the lot testing program after reading the protocol that follows this Commentary should contact the corresponding author.

## Data Availability

Data sharing is not applicable to this article as no datasets were generated or analysed during the current study.
